# Physical Activity Modifies the Association Between Atherogenic Index of Plasma and New-Onset Diabetes in Middle-Aged and Older Adults

**DOI:** 10.3390/healthcare14111529

**Published:** 2026-06-01

**Authors:** Yuhong Song, Jinyan Lan, Yu Ke, Lixu Tang

**Affiliations:** College of Martial Arts, Wuhan Sports University, Wuhan 430079, China

**Keywords:** physical exercise, atherogenic index of plasma, new-onset diabetes, CHARLS

## Abstract

**Highlights:**

**What are the main findings?**

- Elevated atherogenic index of plasma (AIP) is linearly associated with a higher risk of new-onset diabetes.
- The risk of diabetes increases 2.27-fold per one-standard-deviation increase in AIP.

**What are the implications of the main findings?**

- Regular physical activity significantly attenuates the positive association between AIP and diabetes risk.
- The AIP–diabetes link remains potent in non-exercisers but loses significance in regular exercisers.

**Abstract:**

**Background:** While existing research has examined the association between the atherogenic index of plasma (AIP) and the incidence of new-onset diabetes, there is a paucity of evidence concerning the influence of physical activity (PA) on this relationship. This study aims to elucidate the intricate relationships among AIP, PA, and new-onset diabetes in middle-aged and older adults. **Methods:** Data from the 2011–2020 CHARLS cohort were analyzed. Multivariable logistic regression and restricted cubic splines (RCS) assessed the association between AIP and diabetes risk. Effect modification by physical activity was examined via interaction terms and subgroup analyses, with sensitivity analyses excluding participants with dyslipidemia. **Results**: During follow-up, 717 participants developed diabetes. Each one-standard-deviation increase in AIP was associated with a significantly increased risk of diabetes (odds ratio (OR) = 2.266, 95% confidence interval (CI): 1.807–2.843), with a linear dose–response relationship. A significant interaction was observed between AIP and physical activity. The association between AIP and diabetes was robust among non-exercisers (OR = 2.735, 95% CI: 2.087–3.582), but was markedly attenuated and non-significant among regular exercisers (OR = 1.471, 95% CI: 0.962–2.247). Sensitivity analysis yielded consistent results (OR = 2.259, 95% CI: 1.776–2.873). **Conclusions:** AIP is independently associated with an increased risk of new-onset diabetes in Chinese adults aged ≥45 years. Subgroup analyses indicated that physical activity significantly modified this association. The association was robust among non-exercisers but was markedly attenuated and became non-significant among regular exercisers.

## 1. Introduction

As the global population ages rapidly, the incidence of diabetes among middle-aged and older adults is becoming a major public health issue. Diabetes prevalence is expected to increase from 9.3% in 2019 to 10.9% by 2045 [1]. This complex metabolic disorder, influenced by environmental and biological factors, leads to insulin resistance and β-cell dysfunction [2]. The International Diabetes Federation projects that 589 million adults will have diabetes by 2024, rising to 853 million by 2050 [3]. In China, about 11% of adults, or 140 million people, have diabetes, with those aged 65 and older at higher risk [4]. Dyslipidemia significantly contributes to diabetes development [5].

AIP is a novel lipid marker derived from the logarithm of the triglycerides to high-density lipoprotein cholesterol ratio, adjusted for molar concentration [6]. Clinically, this disorder is characterized by high triglycerides (TG) and low high-density lipoprotein cholesterol (HDL-C) levels [7]. Excess free fatty acids (FFAs) lead to insulin resistance by disrupting insulin signaling and causing lipotoxicity [8,9,10], which harms pancreatic β-cells through oxidative stress [11]. HDL-C is vital for its antioxidant and anti-inflammatory effects in managing metabolic disorders like diabetes [12]. Thus, finding strategies to regulate lipid metabolism and improve insulin sensitivity is essential [13].

Physical activity is a cornerstone of type 2 diabetes prevention and management. This cost-effective, non-drug intervention can reduce incidence, improve glycemic control, and enhance overall quality of life [14]. Its benefits are mediated through multiple pathways, including the preservation of β-cell function [15], increased insulin sensitivity [16], improved vascular health [17], and favorable modulation of gut microbiota [18]. Regular exercise also improves body composition and lipid profiles by enhancing skeletal muscle fatty acid oxidation, which lowers TG and raises HDL-C levels. At the molecular level, exercise activates the AMP-activated protein kinase (AMPK) pathway [19,20], promoting the translocation of glucose transporter 4 (GLUT4) to facilitate insulin-independent glucose uptake [21]. Furthermore, it helps mitigate the progression from prediabetes to diabetes by improving glycemic regulation and reducing inflammation and oxidative stress [22].

While prior studies have established a significant association between AIP and diabetes risk, a notable gap remains in understanding whether this association is uniform across different populations. Specifically, it remains unclear whether physical activity alters the strength of the association between AIP and new-onset diabetes. Beyond its established role as a confounder, physical activity is increasingly understood as a potential effect modifier that can alter the magnitude of risk associated with other factors. Empirical studies have demonstrated its effect-modifying influence on associations involving genetic risk, adiposity, and clinical pain [23,24,25]. Mechanistically, since physical activity improves the very lipid profile and insulin resistance that AIP reflects, it is a strong candidate for modifying the AIP–diabetes link. Furthermore, evidence regarding this interaction in the Chinese middle-aged and older adult population—who face unique lifestyle and metabolic profiles—is scarce. Therefore, this study aimed to investigate the effect modification by physical activity on the association between AIP and new-onset diabetes, using a large-scale, longitudinal cohort.

## 2. Methods

### 2.1. Study Participants

This study utilized a longitudinal cohort design based on data from the China Health and Retirement Longitudinal Study (CHARLS) from 2011 to 2020. It began with a national baseline survey in 2011 and conducted follow-up surveys in 2011, 2013, 2015, 2018, and 2020 across 28 provincial regions using multistage stratified sampling. The survey included questions on demographics, lifestyle, and health. Approved by Peking University (IRB00001052-11015), the study ultimately included 19,000 respondents from 12,400 households by 2020 [26].

**Figure 1:**
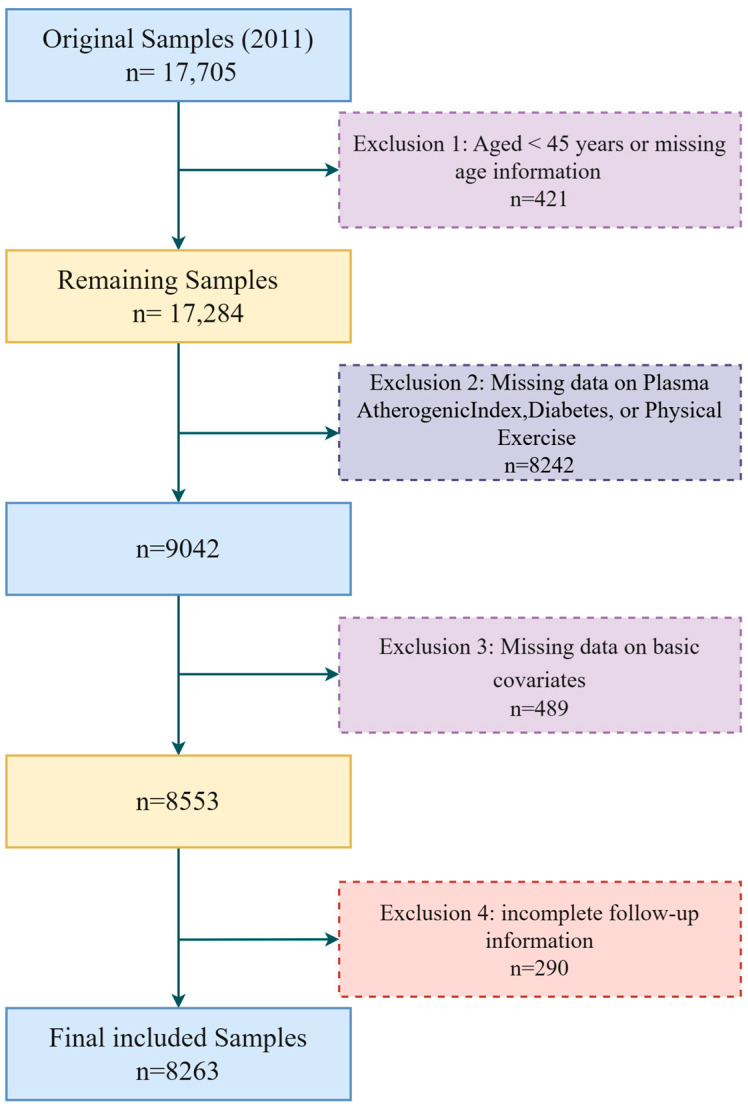
Flowchart of the study.

We utilized data from the 2011 CHARLS cohort. A total of 17,705 participants were initially included in the 2011 baseline survey. To ensure data quality and analytical robustness, we applied the following sequential exclusion criteria (Figure 1):

(1) Exclusion of participants aged <45 years or with missing age information (*n* = 421);
(2) Exclusion due to missing data on Atherogenic index of plasma, Diabetes, or Physical Exercise (*n* = 8242);
(3) Exclusion due to missing data on basic covariates (*n* = 489);
(4) Exclusion due to incomplete follow-up information (*n* = 290).

After applying these inclusion and exclusion criteria (Figure 1), the final analytic sample consisted of 8263 participants.

### 2.2. Assessment of Exposures and Outcomes

The level of physical activity (PA) was assessed using the International Physical Activity Questionnaire (IPAQ) short form within CHARLS. PA was quantified in metabolic equivalent of task (MET) minutes per week (MET-min/week), following the IPAQ scoring protocol. The assessment process was as follows: We collected data on the frequency (days per week) and duration (minutes per day) for four types of activities: vigorous-intensity activity, moderate-intensity activity, walking, and sedentary time. According to the IPAQ guideline, specific MET values were assigned to each activity type: 8.0 METs for vigorous activity, 4.0 METs for moderate activity, and 3.3 METs for walking. For each participant, the weekly MET-minutes for each activity type were calculated as: Frequency (days/week) × Duration (min/day) × Assigned MET value. The total weekly PA (MET-min/week) was the sum of MET-minutes across all three activity types (vigorous, moderate, and walking). Based on the total weekly MET-minutes, participants were categorized into three levels of physical activity intensity: Low (<600 MET-min/week), Moderate (600–3000 MET-min/week), and High (>3000 MET-min/week).

AIP, a key exposure variable, was calculated as the base-10 logarithm of the ratio of fasting triglycerides (TG, in mg/dL) to high-density lipoprotein cholesterol (HDL-C, in mg/dL): AIP = log_10_ (TG/HDL-C). Baseline (2011 wave) measurements were used.

The primary outcome was new-onset type 2 diabetes occurring during the follow-up period (assessed in the 2013, 2015, 2018, and 2020 survey waves). At each follow-up wave, incident diabetes was defined as a participant meeting at least one of the following criteria, provided they were free of diabetes at baseline (2011):

Self-reported diagnosis: A “Yes” response to the question “Have you been diagnosed with diabetes by a doctor?”

Biochemical criteria: Either a measured hemoglobin A1c (HbA1c) level ≥ 6.5% (48 mmol/mol) or a measured fasting plasma glucose (FPG) level ≥ 126 mg/dL [27].

The ascertainment of biochemical criteria was contingent on data availability in each wave. The date of incidence was recorded as the survey wave year when any of the above criteria were first met. Participants were followed from the 2011 baseline until a diabetes diagnosis or the end of the 2020 survey (censoring), whichever occurred first. Key covariates adjusted for in the analyses, measured at baseline, are summarized in Table 1. They include sociodemographic factors, health behaviors, and history of chronic conditions.

### 2.3. Selection of Potential Covariables

Demographic data covers age, gender, marital status, household registration, and education level. Health data includes self-reported smoking, drinking, physical activity, sleep duration, and physician-diagnosed conditions like hypertension, dyslipidemia, chronic lung and kidney diseases, heart disease, and asthma.

### 2.4. Statistical Analyses

All analyses were performed using Stata, version 16.0, StataCorp, College Station, TX, USA. Missing data were handled via complete-case analysis. The Atherogenic Index of Plasma (AIP) was calculated as log10(TG/HDL-C). To reduce the impact of outliers, all continuous covariates were winsorized at the 1st and 99th percentiles prior to analysis.

Normality of continuous variables was assessed using the Shapiro–Wilk test. Since AIP was found to be non-normally distributed (*p* < 0.001), it was presented as median and interquartile range (IQR), and group comparisons were performed using the Mann–Whitney U test. Categorical variables were presented as frequencies (percentages) and compared between groups using the Chi-square test or Fisher’s exact test when appropriate.

Variable selection was based on clinical relevance and existing literature. We constructed a series of four nested multivariable logistic regression models to assess the association of the AIP with incident diabetes, progressively adjusting for potential confounders. The model specifications are as follows:

Model 1: Unadjusted analysis.

Model 2: Adjusted for sociodemographic covariates: age, sex, education, marital status, and residence.

Model 3: Adjusted for covariates in Model 2 plus lifestyle/behavioral factors: smoking status, alcohol consumption, and sleep duration.

Model 4: Adjusted for covariates in Model 3 plus baseline comorbid chronic conditions: hypertension, dyslipidemia, chronic lung disease, kidney disease, heart disease, and asthma.

In sensitivity analyses, AIP was also analyzed in quartiles, with the lowest quartile (Q1) as the reference group. We did not use automated stepwise selection or *p*-value thresholds for variable entry to avoid introducing statistical bias.

## 3. Results

### 3.1. Baseline Characteristics of Participants

Figure 1 illustrates the participant selection process. Of the initial 17,705 records from the 2011 CHARLS wave, 8263 middle-aged and older adults met the inclusion criteria and were included in the final analysis. Table 2 presents the baseline characteristics of the 8263 participants stratified by incident diabetes status. Most participants were married women with an education level of elementary school or lower, primarily residing in rural areas (over 83% with agricultural household registration). Smoking rates differed significantly between groups, with the non-diabetic group having a higher proportion of smokers (39.77%) compared to the new-onset diabetes group (34.31%, *p* = 0.004). In contrast, no significant differences were observed for alcohol consumption (*p* = 0.175) or sleep duration (*p* = 0.291). Regarding physical activity, approximately 36–37% of participants engaged in exercise, mostly at low intensity (32–33%). No significant differences in overall or intensity-specific exercise were found between those who developed diabetes and those who did not (all *p* > 0.05). Individuals with new-onset diabetes exhibited a higher prevalence of comorbidities. Specifically, hypertension was more prevalent in the diabetes group (34.17% vs. 22.12%, *p* < 0.001), as was dyslipidemia (10.46% vs. 7.61%, *p* = 0.007). Differences in chronic lung disease, chronic kidney disease, heart disease, and asthma were not statistically significant (all *p* > 0.05).

### 3.2. The Relationship Between Atherogenic Index of Plasma and New-Onset Diabetes in Middle-Aged and Older Adults

This study used a logistic regression model to analyze the link between the atherogenic index of plasma and new-onset diabetes in middle-aged and older adults. Table 3 details the findings. Model 1 shows unadjusted results, Model 2 adjusts for sociodemographic factors, Model 3 includes health behaviors, and Model 4 adds chronic disease history. The findings reveal that each unit increase in the atherogenic index of plasma significantly raises the risk of new-onset diabetes (OR = 2.266, 95% CI: 1.807–2.843, *p* < 0.05). The study categorized the sample into four groups (Q1–Q4) based on atherogenic index of plasma quartiles. Compared to the Q1 group, middle-aged and older adults in the Q2, Q3, and Q4 groups had progressively higher risks of developing new-onset diabetes, with odds ratios of 1.312, 1.629, and 2.325, respectively, all statistically significant (*p* < 0.05).

### 3.3. Subgroup Analysis of Physical Exercise

Table 4 shows that in middle-aged and older adults, a one-unit rise in the atherogenic index of plasma significantly increases the risk of new-onset diabetes among those who do not exercise (OR = 2.735, 95% CI: 2.087–3.582, *p* < 0.05). However, this risk is not significant in those who exercise (OR = 1.471, 95% CI: 0.962–2.247, *p* > 0.05). Notably, the association remains significant among those participating in low-intensity exercise (OR = 2.618, 95% CI: 2.010–3.409, *p* < 0.05). The interaction *p*-value confirms a significant modifying effect of physical activity on this association.

### 3.4. Sensitivity Analysis

This study excluded middle-aged and older adults diagnosed with dyslipidemia to specifically analyze the association between atherogenic index of plasma and the incidence of new-onset diabetes within this demographic. Table 5 presents the detailed relationship between atherogenic index of plasma and the incidence of new-onset diabetes among middle-aged and older adults, following the exclusion of those with dyslipidemia. The results of the model reveal that each one-unit increase in atherogenic index of plasma is associated with a significant increase in the risk of developing new-onset diabetes in this population (OR = 2.259, 95% CI: 1.776–2.873, *p* < 0.05). These findings are consistent with the results discussed earlier in the study.

### 3.5. Dose–Response

**Figure 2:**
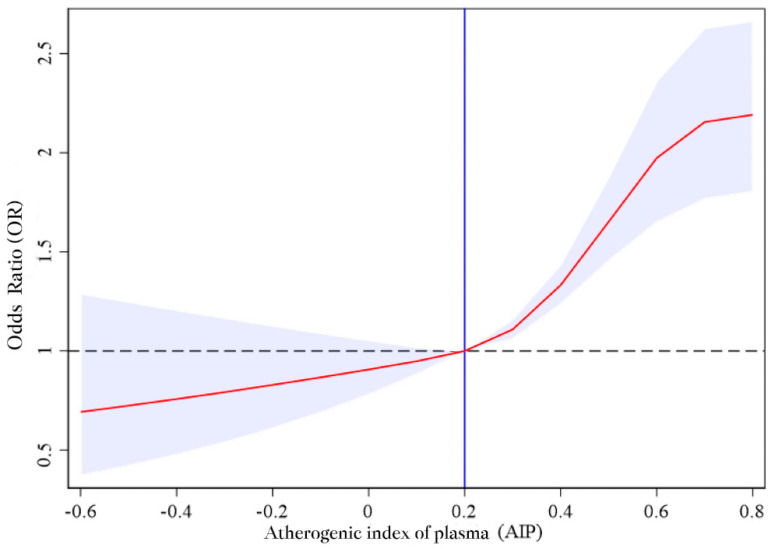
Dose–response relationship between the atherogenic index of plasma (AIP) and the risk of new-onset diabetes. The solid line represents the odds ratio (OR), and the light blue shaded area represents the 95% confidence interval, and the vertical blue line indicates the reference value (AIP = 0.2).

Figure 2 provides a cubic spline model depicting the relationship between AIP and the risk of new-onset diabetes among middle-aged and older adults. The x-axis denotes the AIP, while the y-axis represents the odds ratio (OR) for new-onset diabetes within this demographic. The figure illustrates a discernible upward trend, indicating that as the atherogenic index of plasma rises, there is a corresponding gradual increase in the risk of new-onset diabetes in middle-aged and older adults.

## 4. Discussion

Based on longitudinal cohort data from the CHARLS for the years 2011–2020, this study systematically examined the association between the plasma AIP and the incidence of new onset diabetes among middle-aged and older adults, as well as the moderating and mediating roles of physical exercise in this relationship. The results indicate that for every one-unit increase in AIP, the risk of new onset diabetes among middle-aged and older adults significantly increases (OR = 2.266, 95% CI: 1.807–2.843), and a clear dose–response relationship is observed as AIP quartiles rise.

Subgroup analysis revealed a significant moderating effect of physical exercise (interaction *p* < 0.05). The association was pronounced among individuals who did not engage in physical exercise (OR = 2.735, 95% CI: 2.087–3.582), but was attenuated and not statistically significant among those who did engage in physical exercise (OR = 1.471, 95% CI: 0.962–2.247). Further stratification suggested that this moderating effect may be primarily driven by regular low-intensity exercise. These findings provide new evidence-based support for the early prevention and targeted intervention of diabetes in middle-aged and older adults.

Our findings align with studies showing that elevated AIP reflects systemic metabolic dysregulation, which may drive microvascular injury in the prediabetes stage. Such subclinical vascular damage could further exacerbate metabolic deterioration, creating a vicious cycle that culminates in new-onset diabetes [28]. This study found a significant positive association between AIP and the risk of new onset diabetes in middle-aged and older adults, a finding that is highly consistent with the conclusions of numerous previous studies [4].

A study based on data from the U.S. NHANES 2011–2018 confirmed that AIP is significantly associated with the risk of prediabetes and diabetes in adults, with a stronger association observed among women [29,30]. A multicenter retrospective cohort study in China demonstrated that AIP has significant predictive value regarding the reversal of prediabetes to normal glucose levels or the progression to diabetes [31]. Li et al., using the cumulative AIP (CumAIP) metric derived from the same CHARLS cohort, also found a markedly higher risk in the highest quartile [32]. Under stricter criteria and a longer follow-up period, our study further validates the independent predictive value of AIP, demonstrating a clear dose–response relationship (*p* for trend < 0.001).

Notably, the restricted cubic spline analysis revealed an increasing linear trend between AIP and diabetes risk, with no apparent threshold effect. This aligns with the linear relationship observed by Li et al. but differs from the nonlinear threshold effect identified in some cross-sectional studies [33]. This discrepancy may relate to fundamental differences in study design. Furthermore, studies indicate that the metabolic effects of atherosclerosis may span the entire risk spectrum, exhibiting a linear relationship [34].

From a pathophysiological perspective, AIP is derived from the logarithm of the ratio of triglycerides (TG) to high-density lipoprotein cholesterol (HDL-C) and provides a comprehensive reflection of the lipid microenvironment that contributes to atherosclerosis in the body [35]. Elevated TG levels can impair pancreatic β-cell function through mechanisms such as endoplasmic reticulum stress and inflammation [36,37]. Concurrently, reduced HDL-C may impair insulin receptor integrity and peripheral insulin sensitivity [38,39]. The atherogenic dyslipidemia reflected by the AIP is closely and bidirectionally associated with insulin resistance, form a core pathophysiological basis for new onset diabetes.

The moderating effect of physical exercise observed in this study has significant practical implications. For middle-aged and older adults who may face challenges in sustaining high-intensity exercise, low-intensity activities such as walking and tai chi are more feasible and sustainable [40]. The underlying biological mechanisms through which physical exercise exerts its regulatory effects involve multiple levels. Regular exercise can activate the AMP-activated protein kinase (AMPK) pathway, thereby promoting the expression and translocation of glucose transporter 4 (GLUT4) on the surface of skeletal muscle cells, which in turn enhances glucose uptake and utilization in peripheral tissues and improves insulin sensitivity [41,42]. It can also directly lower plasma triglyceride levels and increase HDL-C concentrations, thereby improving the atherogenic lipid profile reflected by the AIP at its root cause [43,44]. Previous trials have shown that aerobic exercise can significantly reduce AIP levels [45,46]. Additionally, exercise may promote pancreatic beta cell function recovery and reduce systemic inflammation [47,48], collectively mitigating the risk associated with elevated AIP.

In conclusion, this study confirms AIP as an independent risk factor for new onset diabetes in middle-aged and older adults and identifies regular physical exercise, particularly low-intensity activity, as a significant modifier that can attenuate this risk. These results underscore the importance of combining lipid profile management with the promotion of feasible physical activity for diabetes prevention in this population.

## 5. Advantages and Limitations

This study has several strengths. Utilizing the nationally representative longitudinal CHARLS cohort with 8263 participants followed for up to 9 years (2011–2020) provides longitudinal evidence for the association between AIP and new-onset diabetes, an improvement over cross-sectional designs. Furthermore, building on research linking AIP to diabetes, this study explored the moderating role of physical exercise in this relationship within a middle-aged and older adult cohort, analyzing both participation and different intensities of activity. Sensitivity analyses excluding individuals with dyslipidemia yielded consistent results (OR = 2.259, 95% CI: 1.776–2.873), supporting the robustness of the primary finding.

Several limitations should be acknowledged. First, diabetes diagnosis was based on self-report, HbA1c, and fasting blood glucose; the lack of routine oral glucose tolerance tests (OGTT) may have led to some under-ascertainment of cases, and diabetes subtypes could not be distinguished, though the age distribution suggests most were type 2 diabetes. Second, physical activity was self-reported, which is subject to recall and social desirability biases, limiting the precision of activity quantification. Third, despite adjusting for key covariates, data limitations prevented controlling for other potential confounders such as inflammatory markers (e.g., C-reactive protein), dietary patterns, medication use (e.g., statins), and family history of diabetes; residual confounding cannot be ruled out. Regarding analytical methods, this study employed logistic regression rather than time-to-event models (e.g., Cox regression) to assess the association. A primary consideration was that the CHARLS data, in the waves used for this analysis, do not provide the precise date of diabetes onset for all cases, which is a prerequisite for survival analysis. Under this constraint, and given the relatively low cumulative incidence of new-onset diabetes (8.68%) in our cohort—a condition where the odds ratio approximates the risk ratio—logistic regression provides a valid measure of association. Nevertheless, we acknowledge that not accounting for the exact timing of events is a limitation, and future studies with detailed temporal data would benefit from applying survival models to complement our findings. Finally, although we interpret physical activity as an effect modifier based on interaction tests, the observational nature of our data means we cannot definitively establish causality or rule out the possibility of reverse causation (e.g., early metabolic changes influencing activity levels). Thus, the findings should be interpreted as identifying important associations and effect modification, rather than proving causal or preventive effects.

## 6. Conclusions

In conclusion, our analysis establishes an independent association between elevated AIP levels and increased risk of new-onset diabetes in a middle-aged and older cohort. Notably, we observed that this association is modified by physical activity, particularly of low intensity, suggesting a potential interactive role. The primary contributions of this work are (1) the longitudinal confirmation of the AIP–diabetes association, and (2) the generation of a novel hypothesis regarding physical activity as an effect modifier in this pathway. Future mechanistic and interventional studies are warranted to explore causality and the biological basis of this interaction.

## Figures and Tables

**Table 1:** Variable Selection.

Variable Type	Variable Name	Variable Description
Core Variables	New-Onset Diabetes	New-onset diabetes = 1, no new-onset diabetes = 0
Core variable	Physical Activity	Participation = 1, Non-participation = 0
Core Exposure	Atherogenic index of plasma	Logarithm of the triglyceride-to-HDL cholesterol ratio
Socio-demographic covariates	Age	45–59 years = 1, 60–74 years = 2, 75 years and older = 3
	Gender	Male = 1, Female = 0
	Marital Status	Married = 1, Unmarried/Divorced/Widowed, etc. = 0
	Education Level	Elementary school or below = 1, Middle school = 2, High school or above = 3
	Household Registration Status	Non-agricultural household registration = 1, Agricultural household registration = 0
Health Behavior Covariates	Smoking	Smoker = 1, Non-smoker = 0
	Alcohol consumption	Alcohol consumption = 1, No alcohol consumption = 0
	Sleep Duration	Less than 6 h/day = 1, 6–8 h/day = 2, More than 8 h/day = 3
History of chronic diseases Covariates	Hypertension	Present = 1, Absent = 0
	Dyslipidemia	Present = 1, Absent = 0
	Chronic lung disease	Yes = 1, No = 0
	Chronic kidney disease	Yes = 1, No = 0
	Heart disease	Yes = 1, No = 0
	Asthma	Yes = 1, No = 0

**Table 2:** Baseline characteristics of participants by new-onset diabetes status.

Variable	No Diabetes (*n* = 7546)	New-Onset Diabetes (*n* = 717)	*X* ^2^	*p*
AIP	0.32 (0.13, 0.54)	0.45 (0.24, 0.67)		<0.001
Age group, *n* (%)				0.014
45–59 years	4099 (54.32)	407 (56.76)		
60–74 years	2880 (38.17)	282 (39.33)		
≥75 years	567 (7.51)	28 (3.91)		
Sex, *n* (%)			2.398	0.122
Female	4003 (53.05)	402 (56.07)		
Male	3543 (46.95)	315 (43.93)		
Marital status, *n* (%)			2.755	0.097
Married	6618 (87.70)	644 (89.82)		
Others	928 (12.30)	73 (10.18)		
Education, *n* (%)			0.211	0.857
≤Primary	5283 (70.01)	502 (70.01)		
Middle school	1523 (20.18)	148 (20.64)		
≥High school	740 (9.81)	67 (9.34)		
Residence, *n* (%)			0.454	0.500
Agricultural	6315 (83.69)	607 (84.66)		
Non-agricultural	1231 (16.31)	110 (15.34)		
Smoking, *n* (%)			8.182	0.004
No	4545 (60.23)	471 (65.69)		
Yes	3001 (39.77)	246 (34.31)		
Alcohol, *n* (%)			1.841	0.175
No	5064 (67.11)	499 (69.60)		
Yes	2482 (32.89)	218 (30.40)		
Sleep duration, *n* (%)			1.825	0.401
<6 h	2231 (29.57)	229 (31.94)		
6–8 h	4682 (62.05)	428 (59.69)		
>8 h	633 (8.39)	60 (8.37)		
Hypertension, *n* (%)			53.441	<0.001
No	5877 (77.88)	472 (65.83)		
Yes	1669 (22.12)	245 (34.17)		
Dyslipidemia, *n* (%)			7.367	0.007
No	6972 (92.39)	642 (89.54)		
Yes	574 (7.61)	75 (10.46)		
Chronic lung disease, *n* (%)			0.311	0.577
No	6775 (89.78)	639 (89.12)		
Yes	771 (10.22)	78 (10.88)		
Chronic kidney disease, *n* (%)			0.242	0.623
No	7076 (93.77)	669 (93.31)		
Yes	470 (6.23)	48 (6.69)		
Heart disease, *n* (%)			2.363	0.124
No	6730 (89.19)	626 (87.31)		
Yes	816 (10.81)	91 (12.69)		
Asthma, *n* (%)			0.027	0.869
No	7271 (96.36)	690 (96.23)		
Yes	275 (3.64)	27 (3.77)		
Physical activity, *n* (%)			0.523	0.470
No	4807 (63.70)	447 (62.34)		
Yes	2739 (36.30)	270 (37.66)		
High-intensity PA, *n* (%)			0.036	0.849
No	6421 (85.09)	612 (85.36)		
Yes	1125 (14.91)	105 (14.64)		
Moderate-intensity PA, *n* (%)			2.038	0.154
No	5788 (76.70)	533 (74.34)		
Yes	1758 (23.30)	184 (25.66)		
Low-intensity PA, *n* (%)			0.181	0.670
No	5100 (67.59)	479 (66.81)		
Yes	2446 (32.41)	238 (33.19)		

Note: Data are presented as median (interquartile range, IQR) for continuous variables or number (percentage) for categorical variables. Group comparisons were performed using the Mann–Whitney U test for continuous variables and the Chi-square test for categorical variables.

**Table 3:** Relationship between atherogenic index of plasma and new-onset diabetes in middle-aged and elderly individuals.

Variable	Model 1 OR (95% CI)	Model 2 OR (95% CI)	Model 3 OR (95% CI)	Model 4 OR (95% CI)
Per SD	2.521 (2.024–3.141)	2.487 (1.993–3.102)	2.511 (2.012–3.133)	2.266 (1.807–2.843)
Quartiles				
Q1 (ref.)	1	1	1	1
Q2	1.343 (1.044–1.729)	1.333 (1.035–1.716)	1.336 (1.037–1.720)	1.312 (1.018–1.691)
Q3	1.740 (1.367–2.216)	1.725 (1.354–2.197)	1.725 (1.353–2.198)	1.629 (1.276–2.080)
Q4	2.555 (2.032–3.214)	2.526 (2.007–3.180)	2.541 (2.018–3.200)	2.325 (1.840–2.937)
*p* for trend	<0.001	<0.001	<0.001	<0.001

Notes: Model 1: unadjusted. Model 2: adjusted for age, sex, education, marital status, and residence. Model 3: additionally adjusted for smoking status, alcohol consumption, and sleep duration. Model 4: additionally adjusted for hypertension, dyslipidemia, chronic lung disease, kidney disease, heart disease, and asthma.

**Table 4:** Subgroup analysis of physical activity on the association between AIP and new-onset diabetes.

Variable	Group	OR (95% CI)	*p*-Value for Interaction
Physical Activity			
	No-participation	2.735 (2.087–3.582)	<0.001
	Participated	1.471 (0.962–2.247)	
High-intensity			
	No-participation	2.343 (1.841–2.983)	0.142
	Participated	2.045 (1.042–4.013)	
Moderate-intensity			
	No-participation	2.457 (1.908–3.164)	0.089
	Participated	1.740 (1.033–2.926)	
Low-intensity			
	No-participation	2.618 (2.010–3.409)	0.045
	Participated	1.557 (0.994–2.437)	

**Table 5:** Sensitivity analysis of the association between the AIP and incident diabetes after excluding participants with baseline dyslipidemia.

Variable	Total N	Diabetes Samples (%)	OR	95% CI
Per SD	7617	642 (8.43)	2.259	1.776–2.873
Quantiles				
Q1	1969	107 (5.43)	Ref	Ref
Q2	1946	139 (7.14)	1.302	1.002–1.691
Q3	1893	168 (8.87)	1.588	1.233–2.046
Q4	1806	228 (12.62)	2.307	1.810–2.942
*p* for trend			<0.001	<0.001

## Data Availability

The research data in this paper are sourced from the China Health and Retiree Tracking Survey (CHARLS) database from 2011 to 2020. (https://charls.pku.edu.cn (accessed on 18 December 2025)). The survey data were approved by the Ethics Review Committee of Peking University (No. irb00001052-11015).

## Abbreviations

The following abbreviations are used in this manuscript:

CHARLS	China Health and Retirement Longitudinal Study
PA	physical activity
AIP	atherogenic index of plasma
RCS	Restricted cubic splines
HDL-C	high-density lipoprotein cholesterol
TG	triglycerides
FFAs	free fatty acids
AMPK	AMP-activated protein kinase
GLUT4	glucose transporter 4
FPG	Fasting Plasma Glucose

## References

[1] Saeedi P., Petersohn I., Salpea P., Malanda B., Karuranga S., Unwin N., Colagiuri S., Guariguata L., Motala A.A., Ogurtsova K. (2019). Global and regional diabetes prevalence estimates for 2019 and projections for 2030 and 2045: Results from the International Diabetes Federation Diabetes Atlas, 9(th) edition. Diabetes Res. Clin. Pract..

[2] Zhang Y., Wang D.D. (2026). Gut microbiome in type 2 diabetes: Insights from metagenomics, multi-omics, and diet-microbe interactions. Gut Microbes.

[3] Ogle G.D., Wang F., Haynes A., Gregory G.A., King T.W., Deng K., Dabelea D., James S., Jenkins A.J., Li X. (2025). Global type 1 diabetes prevalence, incidence, and mortality estimates 2025: Results from the International diabetes Federation Atlas, 11th Edition, and the T1D Index Version 3.0. Diabetes Res. Clin. Pract..

[4] He X.Y., Lu Z.W., Guo Y.F., Zhao R.C., Liu Z. (2025). Association between atherogenic index of plasma and new onset of type 2 diabetes among elderly in China: A longitudinal study. Front. Endocrinol..

[5] Hill M.J., Metcalfe D., McTernan P.G. (2009). Obesity and diabetes: Lipids, ‘nowhere to run to’. Clin. Sci..

[6] Dobiásová M., Frohlich J. (2001). The plasma parameter log (TG/HDL-C) as an atherogenic index: Correlation with lipoprotein particle size and esterification rate in apoB-lipoprotein-depleted plasma (FER(HDL)). Clin. Biochem..

[7] Boden G., Lebed B., Schatz M., Homko C., Lemieux S. (2001). Effects of acute changes of plasma free fatty acids on intramyocellular fat content and insulin resistance in healthy subjects. Diabetes.

[8] Liu J., Jahn L.A., Fowler D.E., Barrett E.J., Cao W., Liu Z. (2011). Free fatty acids induce insulin resistance in both cardiac and skeletal muscle microvasculature in humans. J. Clin. Endocrinol. Metab..

[9] Choi C.S., Lee F.N., Youn J.H. (2001). Free fatty acids induce peripheral insulin resistance without increasing muscle hexosamine pathway product levels in rats. Diabetes.

[10] Lam T.K., van de Werve G., Giacca A. (2003). Free fatty acids increase basal hepatic glucose production and induce hepatic insulin resistance at different sites. Am. J. Physiol.-Endocrinol. Metab..

[11] Bhatti J.S., Sehrawat A., Mishra J., Sidhu I.S., Navik U., Khullar N., Kumar S., Bhatti G.K., Reddy P.H. (2022). Oxidative stress in the pathophysiology of type 2 diabetes and related complications: Current therapeutics strategies and future perspectives. Free Radic. Biol. Med..

[12] Di Bartolo B.A., Cartland S.P., Genner S., Manuneedhi C.P., Vellozzi M., Rye K.A., Kavurma M.M. (2021). HDL Improves Cholesterol and Glucose Homeostasis and Reduces Atherosclerosis in Diabetes-Associated Atherosclerosis. J. Diabetes Res..

[13] Manell H., Kristinsson H., Kullberg J., Ubhayasekera S., Mörwald K., Staaf J., Cadamuro J., Zsoldos F., Göpel S., Sargsyan E. (2019). Hyperglucagonemia in youth is associated with high plasma free fatty acids, visceral adiposity, and impaired glucose tolerance. Pediatr. Diabetes.

[14] Ried-Larsen M., Johansen M.Y., MacDonald C.S., Hansen K.B., Christensen R., Wedell-Neergaard A.S., Pilmark N.S., Langberg H., Vaag A.A., Pedersen B.K. (2019). Type 2 diabetes remission 1 year after an intensive lifestyle intervention: A secondary analysis of a randomized clinical trial. Diabetes Obes. Metab..

[15] Heiskanen M.A., Motiani K.K., Mari A., Saunavaara V., Eskelinen J.J., Virtanen K.A., Koivumäki M., Löyttyniemi E., Nuutila P., Kalliokoski K.K. (2018). Exercise training decreases pancreatic fat content and improves beta cell function regardless of baseline glucose tolerance: A randomised controlled trial. Diabetologia.

[16] Kirwan J.P., Solomon T.P., Wojta D.M., Staten M.A., Holloszy J.O. (2009). Effects of 7 days of exercise training on insulin sensitivity and responsiveness in type 2 diabetes mellitus. Am. J. Physiol.-Endocrinol. Metab..

[17] Magalhães J.P., Melo X., Correia I.R., Ribeiro R.T., Raposo J., Dores H., Bicho M., Sardinha L.B. (2019). Effects of combined training with different intensities on vascular health in patients with type 2 diabetes: A 1-year randomized controlled trial. Cardiovasc. Diabetol..

[18] Motiani K.K., Collado M.C., Eskelinen J.J., Virtanen K.A., Löyttyniemi E., Salminen S., Nuutila P., Kalliokoski K.K., Hannukainen J.C. (2020). Exercise Training Modulates Gut Microbiota Profile and Improves Endotoxemia. Med. Sci. Sports Exerc..

[19] Yu Y., Zhu G., Zhang Z., Wang H., Zeng L., Xia J., Liu X., Fang C., Liu S., Yang Y. (2025). Exercise ameliorates nonalcoholic fatty liver disease by reducing the IGFBP5 to IGF1 ratio to activate AMPK pathway. Sci. Rep..

[20] Diniz T.A., de Lima J.E., Teixeira A.A., Biondo L.A., Da R.L., Valadão I.C., Silveira L.S., Cabral-Santos C., de Souza C.O., Rosa N.J. (2021). Aerobic training improves NAFLD markers and insulin resistance through AMPK-PPAR-α signaling in obese mice. Life Sci..

[21] Niu Y., Hu S., Zhang Y., Yang J., Zhang J., He R., Chen L., Xu L., Zhao H., Gan B. (2026). Lactate-activated GPR81/FARP1 signaling drives insulin-independent glucose uptake and metabolic control. Cell Res..

[22] Pengyu F., Huiyun X., Lijing G. (2026). HMGB1: A key molecule linking chronic inflammation to complications in type 2 diabetes mellitus and a target for exercise intervention. Front. Endocrinol..

[23] Ahmad T., Chasman D.I., Buring J.E., Lee I.M., Ridker P.M., Everett B.M. (2011). Physical activity modifies the effect of LPL, LIPC, and CETP polymorphisms on HDL-C levels and the risk of myocardial infarction in women of European ancestry. Circ. Cardiovasc. Genet..

[24] Kim K.H., Sami M., Wilson K.S., Fisher K.L. (2025). Physical activity as a moderator of the association between central obesity and Type 2 diabetes in Asian American older adults. Obes. Pillars.

[25] Gyasi R.M., Adjakloe Y., Siaw L.P., James P.B., Amoah P.A., Abass K., Adu-Gyamfi S., Phillips D.R. (2022). The effect-modification of physical activity on the association of pain with impaired physical function in aging adults. Exp. Gerontol..

[26] Zhao Y., Hu Y., Smith J.P., Strauss J., Yang G. (2014). Cohort profile: The China Health and Retirement Longitudinal Study (CHARLS). Int. J. Epidemiol..

[27] American Diabetes Association Professional Practice Committee (2022). 2. Classification and Diagnosis of Diabetes: Standards of Medical Care in Diabetes-2022. Diabetes Care.

[28] Lamprou S., Evangelidis N., Koletsos N., Zografou I., Stoimeni A., Mintziori G., Gkolias V., Trakatelli C.M., Savopoulos C., Doumas M. (2026). Microvascular Dysfunction in Patients with Prediabetes: Novel Methods Identify Impaired Microcirculation. Life.

[29] Shi Y., Wen M. (2023). Sex-specific differences in the effect of the atherogenic index of plasma on prediabetes and diabetes in the NHANES 2011–2018 population. Cardiovasc. Diabetol..

[30] Yin B., Wu Z., Xia Y., Xiao S., Chen L., Li Y. (2023). Non-linear association of atherogenic index of plasma with insulin resistance and type 2 diabetes: A cross-sectional study. Cardiovasc. Diabetol..

[31] Zheng X., Zhang X., Han Y., Hu H., Cao C. (2023). Nonlinear relationship between atherogenic index of plasma and the risk of prediabetes: A retrospective study based on Chinese adults. Cardiovasc. Diabetol..

[32] Li Y., Gu R., Chen L., Zhao Q., Wang Y. (2025). Cumulative atherosclerosis index of plasma exposure and new-onset diabetes in middle-aged and older adults: A prospective cohort analysis from the China Health and Retirement Longitudinal Study. Front. Nutr..

[33] Yang S., Gou X., Dong H., Chen L., Wang Y., Wu J. (2024). Physical activity modifies the association between atherogenic index of plasma and prediabetes and diabetes: A cross-sectional analysis. J. Diabetes.

[34] Kim S.H. (2024). Reframing prediabetes: A call for better risk stratification and intervention. J. Intern. Med..

[35] Niroumand S., Khajedaluee M., Khadem-Rezaiyan M., Abrishami M., Juya M., Khodaee G., Dadgarmoghaddam M. (2015). Atherogenic Index of Plasma (AIP): A marker of cardiovascular disease. Med. J. Islam. Repub. Iran.

[36] Chen J., Fei S., Chan L., Gan X., Shao B., Jiang H., Li S., Kuang P., Liu X., Yang S. (2025). Inflammatory signaling pathways in pancreatic β-cell: New insights into type 2 diabetes pathogenesis. Pharmacol. Res..

[37] Eguchi N., Vaziri N.D., Dafoe D.C., Ichii H. (2021). The Role of Oxidative Stress in Pancreatic β Cell Dysfunction in Diabetes. Int. J. Mol. Sci..

[38] Gursky O. (2015). Structural stability and functional remodeling of high-density lipoproteins. Febs Lett..

[39] Ouyang F.W., Chiang H.H., Hsu W.L., Tsai M.H., Huang C.Y., Remaley A.T., Akyol O., Chen C.H. (2025). Dysfunctional high-density lipoprotein: An updated review. Front. Cardiovasc. Med..

[40] Fridberg H., Wiklund M., Snellman F., Rosendahl E., Hedlund M., Boraxbekk C.J., Lindelöf N. (2025). Negotiating a physically active life in tune with ageing: A grounded theory study of older persons’ experiences of participating in high-intensity interval training. BMC Geriatr..

[41] Friedrichsen M., Mortensen B., Pehmøller C., Birk J.B., Wojtaszewski J.F. (2013). Exercise-induced AMPK activity in skeletal muscle: Role in glucose uptake and insulin sensitivity. Mol. Cell. Endocrinol..

[42] Mingzheng X., You W. (2025). AMPK/mTOR balance during exercise: Implications for insulin resistance in aging muscle. Mol. Cell. Biochem..

[43] Franczyk B., Gluba-Brzózka A., Ciałkowska-Rysz A., Ławiński J., Rysz J. (2023). The Impact of Aerobic Exercise on HDL Quantity and Quality: A Narrative Review. Int. J. Mol. Sci..

[44] Wang X., Huang C., Liu Y., Han Y., Hu H. (2022). Association of estimated glomerular filtration rate and incident pre-diabetes: A secondary 5-year longitudinal cohort study in Chinese people. Front. Endocrinol..

[45] Venojärvi M., Korkmaz A., Wasenius N., Manderoos S., Heinonen O.J., Lindholm H., Aunola S., Eriksson J.G., Atalay M. (2013). 12 weeks’ aerobic and resistance training without dietary intervention did not influence oxidative stress but aerobic training decreased atherogenic index in middle-aged men with impaired glucose regulation. Food Chem. Toxicol..

[46] Shen S., Qi H., He X., Lu Y., Yang C., Li F., Wang L., Qiang D., Shui K., Zhou L. (2018). Aerobic Exercise for a Duration of 90 min or Longer Per Week may Reduce the Atherogenic Index of Plasma. Sci. Rep..

[47] Colberg S.R., Sigal R.J., Yardley J.E., Riddell M.C., Dunstan D.W., Dempsey P.C., Horton E.S., Castorino K., Tate D.F. (2016). Physical Activity/Exercise and Diabetes: A Position Statement of the American Diabetes Association. Diabetes Care.

[48] Jung U.J., Choi M.S. (2014). Obesity and its metabolic complications: The role of adipokines and the relationship between obesity, inflammation, insulin resistance, dyslipidemia and nonalcoholic fatty liver disease. Int. J. Mol. Sci..

